# Subcellular dissemination of prothymosin alpha at normal physiology: immunohistochemical vis-a-vis western blotting perspective

**DOI:** 10.1186/s12899-016-0021-4

**Published:** 2016-03-01

**Authors:** Caroline Mwendwa Kijogi, Christopher Khayeka-Wandabwa, Keita Sasaki, Yoshimasa Tanaka, Hiroshi Kurosu, Hayato Matsunaga, Hiroshi Ueda

**Affiliations:** Department of Molecular Microbiology and Immunology, Division of Immunology, Graduate School of Biomedical Sciences, Nagasaki University, Nagasaki, Japan; African Population and Health Research Center (APHRC), P. O. Box 10787-00100, Nairobi, Kenya; Institute of Tropical Medicine and Infectious Diseases-KEMRI (ITROMID-KEMRI), Nairobi, Kenya; Department of Pharmacology and Therapeutic Innovation, Graduate School of Biomedical Sciences, Nagasaki University, Nagasaki, Japan

**Keywords:** Prothymosin α, Anti-C terminal antibody, Anti- N terminal antibody, Immunohistochemistry, Western blotting and localization

## Abstract

**Background:**

The cell type, cell status and specific localization of Prothymosin α (PTMA) within cells seemingly determine its function. PTMA undergoes 2 types of protease proteolytic modifications that are useful in elucidating its interactions with other molecules; a factor that typifies its roles. Preferably a nuclear protein, PTMA has been shown to function in the cytoplasm and extracellularly with much evidence leaning on pathognomonic status. As such, determination of its cellular distribution under normal physiological context while utilizing varied techniques is key to illuminating prospective validation of its distinct functions in different tissues. Differential distribution insights at normal physiology would also portent better basis for further clarification of its interactions and proteolytic modifications under pathological conditions like numerous cancer, ischemic stroke and immunomodulation. We therefore raised an antibody against the C terminal of PTMA to use in tandem with available antibody against the N terminal in a murine model to explicate the differences in its distribution in brain cell types and major peripheral organs through western blotting and immunohistochemical approaches.

**Results:**

The newly generated antibody was applied against the N-terminal antibody to distinguish truncated versions of PTMA or deduce possible masking of the protein by other interacting molecules. Western blot analysis indicated presence of a truncated form of the protein only in the thymus, while immunohistochemical analysis showed that in brain hippocampus the full-length PTMA was stained prominently in the nucleus whereas in the stomach full-length PTMA staining was not observed in the nucleus but in the cytoplasm.

**Conclusion:**

Truncated PTMA could not be detected by western blotting when both antibodies were applied in all tissues examined except the thymus. However, immunohistochemistry revealed differential staining by these antibodies suggesting possible masking of epitopes by interacting molecules. The differential localization patterns observed in the context of nucleic versus cytoplasmic presence as well as punctate versus diffuse pattern in tissues and cell types, warrant further investigations as to the forms of PTMA interacting partners.

**Electronic supplementary material:**

The online version of this article (doi:10.1186/s12899-016-0021-4) contains supplementary material, which is available to authorized users.

## Background

Prothymosin α (PTMA) is an unstructured, extremely acidic protein (pI 3.5), expressed in a wide variety of cell types. Progressively, research has attested to it as a protein of clinical significance and potential medical use [1, 2]. It is considered a nuclear protein with a potent nuclear localization signal (NLS), albeit reports indicate cytoplasmic and extracellular presence as well, under specific physiological or pathological conditions [3, 4]. It has a highly conserved amino acid sequence among mammals and this together with its wide distribution affirm that it plays an essential role in an organism other than the traditionally attributed role of immune modulation.

Even so, the precise function is still not well understood. Accumulating data point towards both intracellular and extracellular biological functions of PTMA [5–11]. The major intracellular role is associated with cell proliferation. It has been reported that PTMA stimulates cell proliferation by sequestering a repressor of estrogen receptor activity in various cells [12]. Cancer cells are known to be highly proliferative and indeed high expression of intracellular PTMA has been correlated with progression of a number of cancer types including bladder cancer, liver cancer, breast cancer, head and neck cancer, lung cancer, colon cancer, rectal cancer, ovarian cancer, neuroblastoma, prostate cancer and gastric cancer [13–26]. Moreover, PTMA binds to histones, p300 histone acetyl transferase and cAMP response element binding protein (CREB)-binding protein to induce gene transcription [27, 28]. Recent studies have explored the role of intracellular PTMA in acetylation regulation [29].

Further intracellular roles of PTMA include its involvement in cell survival. Nuclear Factor erythroid 2 related factor (Nrf2), a transcription factor that regulates expression of defensive genes such as antioxidant proteins and detoxifying enzymes, is inhibited by Kelch-like ECH-associated protein 1(Keap1). On one hand, PTMA binds to Keap1 to dissociate Keap1-Nrf2 complex thus upregulating the expression of Nrf2 dependent anti-oxidative stress genes [30]. On the other hand, it has been reported in mediating nuclear import of Keap1 to degrade nuclear Nrf2 and thus switch off downstream gene expression suggesting that it is involved in on/off switch of Keap1-Nrf2 system [31]. Moreover, PTMA acts as an anti-apoptotic molecule in the cytoplasm by interacting with Apaf1 to inhibit apoptosome formation [32] and by binding to cytochrome c [33] thereby inhibiting caspase activation. Reports also show that it interacts with p8 (nuclear protein 1), a natively unstructured protein with anti-apoptotic activity much like PTMA, to form a heterodimer complex that could act in concert to regulate the apoptotic cascade [34]. Interestingly however, PTMA undergoes caspase mediated fragmentation in apoptotic cells at two amino-terminal sub-optimal sites and one carboxy-terminal optimal site [35, 36]. Cleavage at the latter site disrupts its nuclear localization and subsequently its intranuclear functioning [37].

Extracellularly, PTMA plays a role in immunomodulation via a pleiotropic mode by stimulating a variety of immune cells including natural killer cells, T cells and lymphokine-activated killer cells-dendritic cells, monocytes and macrophages. The mechanism by which it regulates immune responses is proposed to be through binding to TLR4 on innate immune cells to trigger induction of pro-inflammatory cytokines. Reports also show that the C-terminus of PTMA holds the immunologically active site of the polypeptide [38]. Seemingly, different parts of the molecule are responsible for the different biological functions it exerts.

Reports from our laboratory also show that extracellular PTMA can confer protection to neurons upon ischemic stress by inhibiting necrosis [39]. Cell death that occurs as a consequence of obstructed blood flow in a brain region causes damage to tissues and results in ischemic stroke. Two modes of cell death occur during ischemic stroke: necrosis at the ischemic core and apoptosis several days later at the region that surrounds the core called the penumbra. Necrosis, a rapid and expanding process contributes largely to cell damage but to this end there are no known inhibitors of necrosis. Apoptosis on the other hand can be inhibited by growth factors. Importantly, PTMA was identified as a molecule that can initiate a switch from uncontrollable necrosis to tractable apoptosis in ischemic neurons [40]. Further findings on the neuroprotective role of PTMA revealed neuron-specific extracellular release of the polypeptide following ischemic stress. PTMA was released in a non-classical manner and this release was mediated by its interaction with the cargo protein, S100A13 [41]. The interaction was shown to require the C-terminal region of PTMA. Since astrocytes have intrinsic caspase 3 activity it was postulated that the non-release of PTMA in astrocytes in brain following ischemia was as a result of caspase cleavage of PTMA in its C-terminus thus depriving it the interaction with S100A13. In addition, caspase cleavage of PTMA disrupts its nuclear localization, resulting in redistribution of the polypeptide. PTMA is also capable of proteolytic lysis by asparaginyl endopeptidase in its N-terminus to yield thymosin alpha 1 (TA_1_) but it still remains to be elucidated if this proteolysis is simply a step in the catabolism of PTMA or a more specific selective process in view of some biological function of TA_1_ [42].

Depending on the proteins which it interacts with, PTMA exerts different effects; a feature that is determined by its subcellular localization. To individualize PTMA interactions and proteolytic modifications that occur in different tissues, it is a prerequisite to determine its cellular modelling in diverse tissues. The subcellular localization in tissues under normal physiological context has been under reported yet this is a core basis for further elucidation of its interactions and proteolytic modifications under pathological conditions like the various cancer forms, ischemic stroke and immunomodulation mechanisms where it exhibits extensively contrasting roles depending on the pathognomonic status and localization. In the present study, we implore western blotting and immunohistochemical techniques to explore the expression of PTMA and its specific cellular localization in different murine tissues using a set of monoclonal antibodies raised against different epitopes on the polypeptide; the brain being the predominant organ of investigation based on our earlier findings [39–41]. First, we generated a monoclonal antibody against the C-terminal region of PTMA (AntiCT) and employed it in conjunction with the anti N-terminal monoclonal antibody (2 F11) for a comparison. We therefore observe that, the preferential combination of the applied techniques and monoclonal antibodies would lay ground to prospective examinations for better understanding of factors that mediate biological function of PTMA across brain cells and other peripheral tissues not only during proteolytic modifications in pathological conditions but also under normal physiology context.

## Methods

### Experimental animals

Two female Wistar rats were used for the generation of monoclonal antibodies. Male MP-BL mice were used for the western blotting and immunohistochemistry experiments. The animals were kept in a room maintained at constant temperature (21 ± 2 °C) and relative humidity (55 ± 5 %) with an automatic 12 h light/dark cycle with free access to standard laboratory diet and water *ad libitum*.

### Immunization of animals

Synthetic biotinylated carboxy-terminal peptide of PTMA, (bearing the sequence DDVDTKKQKTEEDD, -Cat.No 293211. GL Biochem Shanghai Ltd), was used as immunogen. Wistar rats were anesthetized and immunized with 200 μg avidin conjugated immunogen, injected on the foot pads as emulsions (1:1 v/v) in complete Freund’s adjuvant (Cat No. 263810, DIFCO Laboratories Detroit, MI) Blood was collected from the immunized animals 2 weeks after the injection and the serum tested for immune reactivity with recombinant mouse PTMA. A week later the animals were sacrificed and the medial iliac lymph nodes aseptically harvested as previously reported [43, 44].

### Characterization of the antibody

Hybridomas were prepared using the isolated medial iliac lymph node cells and SP2/0 myeloma cells as the fusion partner in the presence of polyethylene glycol (PEG) and cultured in Hypoxthanthine Aminopterin Thymidine (HAT) selective medium. Cell culture media was then assayed by standard Enzyme linked Immuno-sorbent Assay (ELISA) for the presence of PTMA-reactive antibodies and cells from the ELISA-positive wells cloned by limiting dilution. Screening of hybridoma cell line supernatants was performed via ELISA. Positively identified hybridoma clones were expanded and inspected for monoclonality then assayed by ELISA for immune-reactivity. Desired clones were subsequently re-expanded and adapted to RPMI1640 medium. The monoclonal antibody was then purified from the supernatant by Protein G affinity chromatography and the concentration of purified antibody was determined by Lowry method.

### Application of antibody in western blot analysis

Mice were decapitated and the desired brain regions (Olfactory bulb, cortex, amygdala, hippocampus, striatum, cerebellum, thalamus, hypothalamus, mid brain pons and medulla) and tissues (peripheral tissues including spleen, thymus, lung, heart, kidney, liver, colon, stomach, duodenum, jejunum, ileum and cecum) were obtained and transferred into ice-cold homogenizing buffer (20 mM Tris-HCI, pH 7.4, 10 mM NaCl, 1 mM EDTA, 0.01 % SDS, 1 % Triton X-100 and 1X protease inhibitor cocktail) . Tissue lysis was performed using tissue homogenizer or sonication. The homogenate was centrifuged at 15,000 rpm for 30 min at 4 °C. An aliquot of the supernatant was taken for protein quantification, equivalent amounts of total protein mixed with 2 x SDS sample buffer and heated in a boiling water bath for 5 min. The boiled samples were either used immediately or frozen at –20 °C.

Protein quantification of recombinant PTMA and total protein concentration of tissue homogenates was determined by Lowry protein assay method. Samples were prepared for loading onto gel using 1^×^SDS loading buffer as previously described. Indicated quantities of recombinant Prothymosin α and the tissue samples containing 20 μg of total protein were electrophoresed on 15 % SDS-polyacrylamide gels, transferred onto a nitrocellulose membrane by electroblotting performed at 30 V, 100 mA for 90 min and subjected to immunoblotting with appropriate antibody. In the case of untagged recombinant and tissue PTMA, transfer onto a membrane was by electroblotting with acidic buffer (20 mm sodium acetate buffer, pH 5.2) followed by fixation with 0.5 % glutaraldehyde. Blotted membranes were blocked with 5 % skim milk and 2 % FBS in TBST or 5 % BSA. Membranes were then probed with rat/mouse anti-PTMA (AntiCT and 2 F11 from Alexis Biochemicals respectively) and mouse anti-GAPDH (internal control) primary antibodies followed by Horseradish Peroxidase (HRP)-conjugated goat secondary antibodies. Visualization of immunoreactive bands was executed by enhanced chemiluminescent (ECL). Densitometry of the detected protein bands was determined using ImageJ software. The densitometry ratio of PTMA to GAPDH was calculated. For the purpose of comparison between tissues and between different sets of experiments, the densitometry ratio of each tissue was plotted.

### Application of antibody in immunoprecipitation assay

10 μg Protein G sepharose fast flow slurry was incubated with 10 μg of indicated antibody at 4 °C overnight in agitating condition. The beads were then washed 3 times each with wash buffer and incubated with sample (tissue lysate or recombinant protein), at 4 °C for 4 h followed by 3 washes and eluted with 1X SDS loading buffer. Immunoprecipitated samples were then boiled for 5 min and analysed by western blot.

### Application of antibody to immunohistochemistry

Mice peripheral tissues and specific brain regions were fixed by vascular perfusion with 30 mL of 4 % paraformaldehyde (PFA) after perfusion with PBS for about 20 min. Desired organs were isolated and washed with PBS then processed using a rapid microwave automatic histo-processor using a standard protocol and embedded into paraffin wax. Subsequent immunohistochemistry assays were then performed per standard protocols previously described [15]. Briefly, sectioned tissues were deparrafinized then permeabilized with methanol. The sections were then blocked with 3 % BSA for 1 h at room temperature and later incubated with primary antibody (1:300 mouse monoclonal IgG 2 F11; Enzo Life Sciences Int., PA, USA and AntiCT)) at 4 °C overnight, washed with PBST then with secondary antibody conjugated to AlexFluor (1:600) for 2 h at room temperature. Counterstaining with Hoechst 33342 (1:10,000) was effected. The slides were then mounted by pristine mount and left to dry overnight at 4 °C. Images were collected by BZ-8000 microscope (KEYENCE, Osaka, Japan) with a × 20 Plan APO lens (Nikon, Tokyo, Japan).

### Application of antibody to immunocytochemistry

Primary cortical cells on an eight-well Lab Tek chamber were fixed in 4 % PFA in PBS at 4 °C overnight followed by 3 wash steps with PBS. Fixed cells were permeabilized with 50 and 100 % methanol for 10 min each then washed with PBS 3 times. The permeabilized cells were washed with 0.1 % Triton X-100 in PBS (PBST), incubated in blocking buffer (3 % BSA in PBST) for 3 h at 4 °C, and then incubated in primary antibody (1 : 300; mouse monoclonal IgG 2 F11; Enzo Life Sciences Int., PA, USA and AntiCT) overnight at 4 °C. The cells were then washed with PBST and thereafter incubated in a secondary antibody conjugated to AlexFluor. The nuclei were visualized with Hoechst 33342. Images were collected using a BZ-8000 microscope (KEYENCE, Osaka, Japan) with a × 20 Plan APO lens (Nikon, Tokyo, Japan).

### Ethical clearance

All the procedures were formally approved by Nagasaki University Animal Care Committee. The guidelines were strictly adhered to during the research.

## Results

**Fig. 1:**
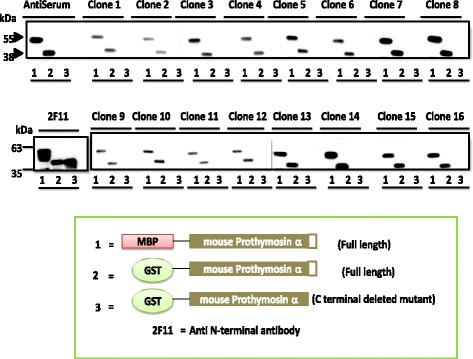
Western blot analysis of hybridoma immune-reactivity MBP and GST tagged recombinant mouse PTMA (as shown in the inlet) were loaded onto gel for electrophoresis and thereafter blotted on to nitrocellulose membranes. Blotted membranes were incubated with supernatant from the hybridoma cultures from different clones as indicated. HRP conjugated anti-rat IgG secondary antibody and HRP substrate was used to develop the bands. Signals were detected by enhanced chemiluminescence. No band was detected on the C-terminal deleted mutant of PTMA (lane 3), while bands corresponding to full-length tagged recombinant PTMA were detected with different intensities across the clones

Selection of hybridoma cell lines secreting monoclonal antibodies by indirect ELISA was performed using purified Glutathione S- transferase (GST) and Maltose binding protein (MBP) tagged recombinant PTMA as the antigen. From approximately 1500 primary hybridomas 16 yielded immune reactive signals 3-100-fold above background as indicated by optical density (OD) values (data not shown). The 16 hybridomas were all confirmed to react with recombinant PTMA in a second round of ELISA screening. To determine whether the secreted antibodies could specifically detect full-length PTMA, the 16 hybridoma candidates were tested for their reactivity against recombinant mouse PTMA by western blot analysis (Fig. 1). The C-terminal truncated PTMA was included to assay the specific detection of C-terminus region. Supernatant from the hybridoma cell culture was used for the analysis. The antibodies from the cultures could detect full-length PTMA but not the C-terminal truncated one.

**Fig. 2:**
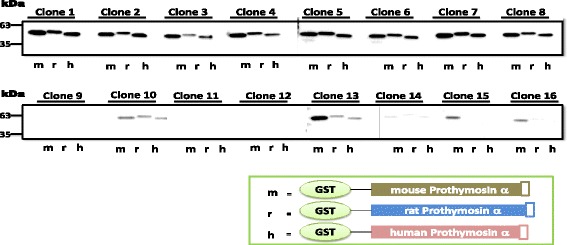
Determination of cross reactivity of the antibodies with rat and human PTMA. GST tagged recombinant mouse, rat and human PTMA were loaded onto gel for electrophoresis and blotted on to nitrocellulose membranes thereafter. Blotted membranes were incubated with supernatant from the hybridoma cultures from different clones as indicated. Antibodies from most clones showed crossreactivity with rat and human PTMA. Some showed relatively similar or different signal intensities indicative of the affinity of the antibodies to the particular antigens. Clones 9, 11 and 12 lost their ability to secret antibodies

**Fig. 3:**
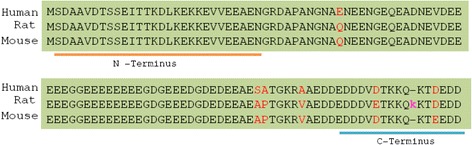
Alignment of amino acid sequences of human, rat and mouse Prothymosin alpha

To further investigate if the generated antibody could detect rat and human PTMA, western blot analysis was carried out using purified recombinant full-length mouse, rat and human Prothymosin α (Fig. 2). Supernatant from the hybridoma culture was used for this analysis. Clones 1, 5 and 7 showed somewhat similar signal intensities for the detection of PTMA across the species. On the other hand, clones 3, 4, 8, 13, 15 and 16 showed higher affinity for mouse PTMA whereas clones 9, 11 and 12 lost their ability to secret the antibodies. Clones 10 and 14 showed relatively very weak signal intensities. PTMA sequence is highly conserved across species (Fig. 3), coloured amino acids denote less conserved sections. While amino termini of all three species show precise homology, the carboxy termini show some differences between the species. Nevertheless, some antibodies from various hybridoma cell lines could recognize the c-terminus of PTMA across species.

Clone 7 (Figs. 1 and 2) was selected for further culture due to its consistency in PTMA detection after cloning. The selected clone was adapted to RPMI1640 medium and antibody immunoreactivity confirmed. The hybrodoma clone was then grown in serum free GIT medium and the secreted antibodies were purified by protein G affinity chromatography, dialyzed with PBS and later antibody concentration was determined. Reactivity of the purified antibody was assayed by western blotting and antibody tested for capacity to immunoprecipitate PTMA from tissue and the recombinant PTMA. AntiCT antibody successfully immunoprecipitated PTMA from the cerebellum tissue lysate. Anti N-terminal PTMA (2 F11) was applied alongside AntiCT in the immunoprecipitation studies for a comparison. Detection of PTMA immunoprecipitated by both antibodies was carried out by 2 F11. Same amount of antibody was used for each antibody type. 2 F11 antibody showed greater capacity for immunoprecipitation than AntiCT.

**Fig. 4:**
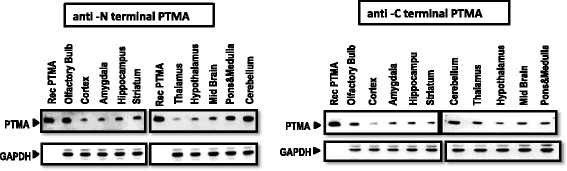
Western blot analysis of the distribution of PTMA in selected brain regions. Immunoreactive PTMA from regions of the brain tissue shows single bands at apparent molecular weight of 12 kDa. Lysates from multiple sections of the brain were resolved in electrophoretic gel and transferred to nitrocellulose membranes. The western blot analysis was performed with monoclonal 2 F11 and monoclonal AntiCT. Untagged recombinant PTMA was used as positive control and GAPDH as internal control

**Fig. 5:**
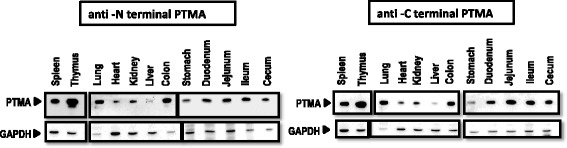
Western blot analysis of the distribution of PTMA across peripheral tissues. Immunoreactive PTMA from various peripheral tissues shows single bands at apparent molecular weight of 12 kDa. Lysates from the peripheral tissues were resolved in electrophoretic gel and transferred to nitrocellulose membranes. The western blot analysis was performed with monoclonal 2 F11 and monoclonal AntiCT. GAPDH was used as internal control

The tissue-wide distribution of PTMA was assessed using the newly generated anti C-terminal PTMA (AntiCT) and the anti N-terminal PTMA (2 F11) antibodies. To explore whether proteolytically modified forms of PTMA could be detected by the set of antibodies, the immunoblot assay was performed in brain regions and peripheral tissues. Immunoblot data revealed a single band detectable by both 2 F11 and AntiCT that corresponded to full-length PTMA in the brain regions (Fig. 4), no additional bands were detected indicative of proteolytic modification of PTMA. In peripheral tissues, a similar observation was made (Fig. 5), however an additional lower band was detectable by 2 F11 only in the thymus suggesting c-terminal truncated version o or several different variants of PTMA could be present. The relative levels of PTMA in different tissues were calculated according to densitometry measurements and the data are summarized. Densitometric quantification of the western blot bands of PTMA expression across peripheral tissues and selected brain regions [Additional files 1 and 2 correspondingly] corresponding to Figs. 4 and 5 respectively. In the brain, the highest levels of PTMA were detected in the olfactory bulb and cerebellum by both antibodies while in peripheral tissues, the thymus, spleen and lungs showed high PTMA levels by both antibodies. Relatively low PTMA levels were detected in the heart, kidney and liver.

**Fig. 6:**
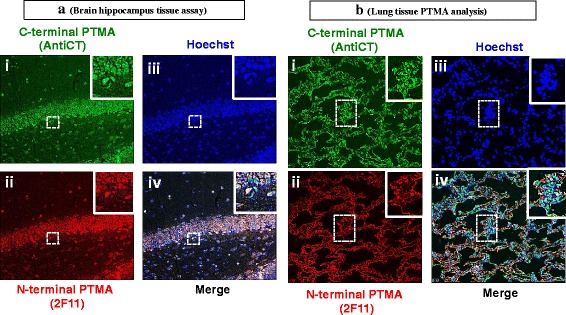
Immunohistochemistry analysis of PTMA in (**a**) mouse brain hippocampus tissue and (**b**) Lung tissue. Whole animal vascular perfusion fixation was conducted with PFA and the required tissues were isolated. The brain hippocampus and lung were processed for immunohistochemistry by embedding to paraffin wax. Tissue sections were stained with AntiCT (a)i and (b)i and 2 F11 (a)ii and (b)ii together with relevant fluorescent tagged antibodies. Sections were then washed, counterstained with Hoechst (a)iii and (b)iii and processed for fluorescence microscopy. Both antibodies localized PTMA in the nucleus of the hippocampus (a)i and ii, while in the lung PTMA immunoreactivity was observed in the nucleus and cytoplasm by AntiCT (b)i but mainly in the cytoplasm by 2 F11 (b)ii

PTMA localization was evaluated by immunohistochemistry in a spectrum of organs Multi-colour experiment was performed for PTMA visualization by co-staining with AntiCT and 2 F11 antibodies. Hoechst stain was used to visualize the nucleus. We observed a differential staining pattern among the tissues examined. Immunoreactivity of PTMA was mainly in the nucleus of most tissues, while other tissues indicated predominant cytoplasmic staining. In the hippocampus PTMA signals were intense in the nucleus (Fig. 6a iii) when detected by both Anti CT and 2 F11 (Fig. 6a i and ii) clearly indicated by the merge data (Fig. 6a iv). In the lungs, an intriguing observation was made in the pattern of distribution of PTMA expression. The AntiCT antibody stained both nucleus and cytoplasm (Fig. 6b i iii and iv) in majority of the cells while 2 F11 stained only the cytoplasm in most cells and the perinuclear space in few cells (Fig. 6b ii, iii and iv).

**Fig. 7:**
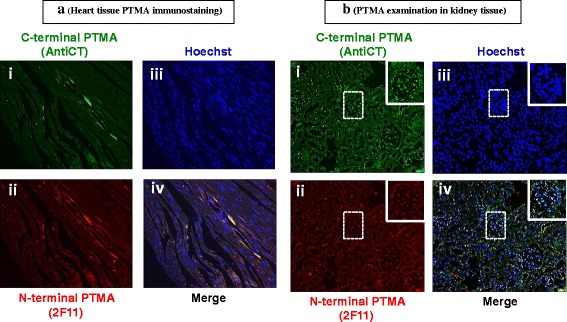
Immunohistochemical examination of PTMA in (**a**) mouse heart and (**b**) kidney tissue. Whole animal vascular perfusion fixation was conducted with PFA and the required tissues were isolated. The heart and kidney were processed for immunohistochemistry by embedding to paraffin wax. Tissue sections were stained with AntiCT (a)i and (b)i and 2 F11(a)ii and(b)ii together with relevant fluorescent tagged antibodies. Sections were then washed, counterstained with Hoechst (a)iii and(b)iii) and processed for fluorescence microscopy. The heart PTMA immunoreactivity was moderate by both antibodies (a)i and ii. The kidney AntiCT showed higher immunofluorescent intensity in comparison to 2 F11 signals in the Kidney (b)i and ii

The heart presented weak PTMA immunostaining by both AntiCT and 2 F11 antibodies (Fig. 7a i, ii and iii) and clearly illustrated by the merge (Fig. 7a iv) in correlation with the western blot data that indicated weak PTMA expression. In the kidney, PTMA was primarily expressed in the nucleus (Fig. 7b iii) by both antibodies (Fig. 7b i and ii) with AntiCT showing more intense signal (Fig. 7b iv).

**Fig. 8:**
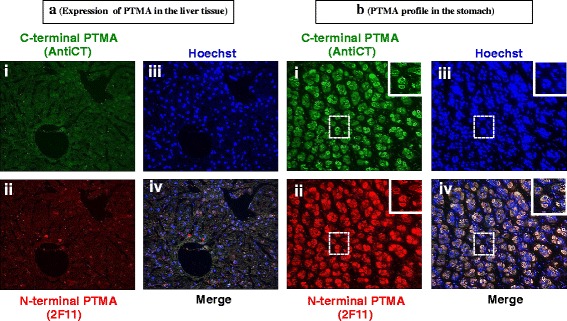
Immunohistochemical examination of PTMA expression levels in (**a**) mouse liver tissue and (**b**) stomach. Whole animal vascular perfusion fixation was conducted with PFA and the required tissues were isolated. The liver and stomach were processed for immunohistochemistry by embedding to paraffin wax. Tissue sections were stained with AntiCT (a)i and (b)i and 2 F11 (a)ii and (b)ii together with relevant fluorescent tagged antibodies. Sections were then washed, counterstained with Hoechst (a)iii and (b)iii and processed for fluorescence microscopy. The liver shows substantial nucleic PTMA immunoreactivity by both antibodies (a)i and ii whereas only the cytoplasm showed immunofluorescence by both 2 F11 and AntiCT in the stomach (b)i and ii

In the liver PTMA was demonstrated considerably in the nucleus (Fig. 8b iii) by both AntiCT ant 2 F11 (Fig. 8a i, ii and iv), this correlates with the western blot data that showed substantial expression of PTMA. In the stomach, PTMA immunoreactive cells of the mucosal layer demonstrated quite a unique distribution pattern. PTMA was not stained in the nucleus (Fig. 8b iii) of these cells by either of 2 F11 or AntiCT antibodies (Fig. 8b i and ii). Interestingly, only the cytoplasm was positively immunostained, clearly indicated by the merge data (Fig. 8b iv). Showing both antibodies exhibited strong cytoplasmic immunofluorescent intensity.

**Fig. 9:**
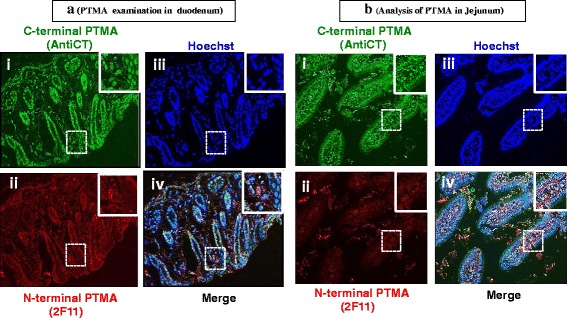
Immunohistochemical analysis of PTMA in (**a**) mouse duodenum and (**b**) Jejunum. Whole animal vascular perfusion fixation was conducted with PFA and the required tissues were isolated. The duodenum and jejunum were processed for immunohistochemistry by embedding to paraffin wax. Tissue sections were stained with AntiCT (a)i and (b)i and 2 F11 (a)ii and (b)ii together with relevant fluorescent tagged antibodies. Sections were then washed, counterstained with Hoechst (a)iii and (b)iii) and processed for fluorescence microscopy. In the duodenum, PTMA immunoreactivity revealed nucleic staining with AntiCT (b)i and cytoplasmic staining with 2 F11 (b)ii

The duodenum showed PTMA immunoreactivity in the nucleus (Fig. 9a iii) and weakly in the cytoplasm but AntiCT (Fig. 9a i) demonstrated higher signal intensity than 2 F11 (Fig. 9a ii). Additionally, 2 F11 depicted perinuclear staining in some submucosal layer cells. The jejunum also presented an exceptional PTMA staining pattern in which the AntiCT shows immunoreactivity mainly in the nucleus (Fig. 9b i and iii) while 2 F11 depicts mainly a cytoplasmic immunostaining (Fig. 9b ii). Some cells show perinuclear staining with 2 F11 only, while few show perinuclear staining with both antibodies (Merge, Fig. 9b iv).

**Fig. 10:**
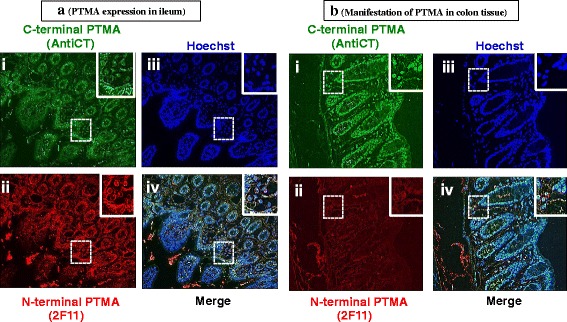
Immuohistochemical analysis of PTMA in (**a**) mouse ileum tissue and (**b**) colon. Whole animal vascular perfusion fixation was conducted with PFA and the required tissues were isolated. The ileum and colon were processed for immunohistochemistry by embedding to paraffin wax. Tissue sections were stained with AntiCT (a)i and (b)i and 2 F11 (a)ii and(b)ii together with relevant fluorescent tagged antibodies. Sections were then washed, counterstained with Hoechst (a)iii and(b)iii and processed for fluorescence microscopy. The ileum and colon depict nucleic PTMA staining with AntiCT (a and b)i and cytoplasmic staining with 2 F11 (a and b)ii

**Fig. 11:**
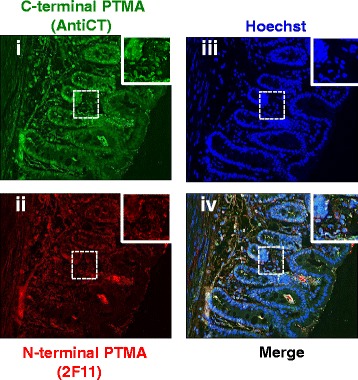
Immuohistochemical examination of PTMA in cecum. The cecum was processed for immunohistochemistry by embedding to paraffin wax. Tissue sections were stained with AntiCT (i) and 2 F11 (ii) together with relevant fluorescent tagged antibodies. Sections were then washed, counterstained with Hoechst (iii) and processed for fluorescence microscopy. Some cells showed PTMA immunoreactivity in both nucleus and cytoplasm with both antibodies while others show nucleus staining with AntiCT and cytoplasmic staining with 2 F11 (i and ii)

Ileum, colon and cecum demonstrated a similar pattern of distribution of immunoreactive PTMA (Figs. 10a, b and 11 respectively). The AntiCT stained with much higher fluorescent intensity (Figs. 10a i, iii; b i, iii and Fig. 11 i, iii), depicting strong nucleus signal compared to cytoplasmic signal. 2 F11 showed moderate nucleus signal (Figs. 10a ii, iii; b ii, iii and Fig. 11 ii, iii). An overlay of PTMA and nuclear staining is shown in the merge data (Figs. 10a, b and 11iv).

**Fig. 12:**
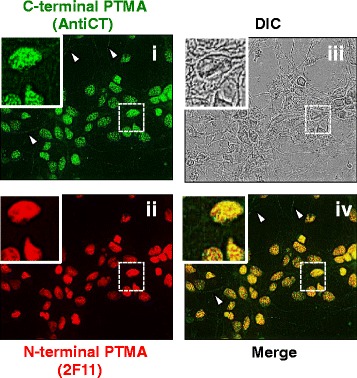
Cellular distribution of PTMA in rat primary neurons. Fixed rat primary cortical neuron cells were permeabilized with 50 and 100 % methanol then washed with PBS. The permeabilized cells were incubated in blocking buffer, and then with AntiCT (a)i and 2 F11(b)ii. Stained cells were then processed for fluorescence microscopy. C-terminal antibody stained PTMA in the primary cortical neurons with a punctate pattern while anti N-terminal showed diffuse nuclear staining. Arrowheads denote the weak cytoplasmic signals detected by the anti C-terminal PTMA antibody only

Given that PTMA has previously been shown by 2 F11 to be exclusively located in the nuclei of neurons we explored cell-type specific distribution of PTMA: an immunocytochemical study, by immunostaining with newly generated anti C-terminus PTMA antibody whether a similar staining pattern would be observed. We used primary cortical neurons cultured from 17 day-old embryonic rat brain. Anti C-terminal PTMA depicted a punctate signal that was widely distributed throughout the nucleus; weak signals were also detected in the cytoplasm as by the arrow heads shown (Fig. 12), eliciting contrast with the 2 F11 antibody.

## Discussion

Prothymosin α (PTMA) generates a great deal of interest among investigators due to its robust involvement in a variety of biological processes ranging from cell proliferation as observed in several types of cancer [13–26] and apoptosis [34, 37, 39, 40, 45] to its association with cell-mediated immunity [6, 8, 46]. Moreover, going by recent studies conducted to understand the role PTMA in non-mammalian vertebrates, insights into changes in PTMA functions and expression that have occurred with evolution is required. It has been shown that PTMA is involved in spermatogenesis in the frog [47, 48] and in the cartilaginous fish *Torpedo marmorata* [49]. In the zebrafish, the PTMA gene has been shown to be duplicated [50] and expressed differentially during embryonic development indicating that their function is more complex in fishes than in mammals.

As yet, its subcellular localization in tissues under normal physiological context has been underreported equally utilizing narrow range techniques. In the present study we demonstrated the tissue distribution and subcellular localization of PTMA in diverse murine organ tissues and cell types. The insights into its differential dissemination in brain cell types and major peripheral organs at normal physiology, would portent better basis for further elucidation of its interactions and proteolytic modifications under pathological conditions. This considers the fact that, it exhibits extensively contrasting roles depending on the pathognomonic status and localization as either intracellular or extracellular. To illuminate the differential distribution and localization of PTMA, with respect to different tissues and cell types, antibodies that discriminate the different epitopes on the polypeptide were useful tools. First we developed an antibody against the C-terminus of PTMA, a region that presents relatively high hydrophilicity and mobility indices and is therefore likely to have high antigenicity, according to a previous theoretical evaluation [51]. Additionally, antigenic determinants of polypeptides are usually located in their N and/or C termini therefore the C-terminus of PTMA is putatively immunogenic. The rat iliac lymph node method was utilized to produce the monoclonal antibodies instead of the conventional spleen method as it offers a number of benefits; (a) a single injection is sufficient for immunization, (b) lymph nodes are ready to use 2 weeks after injection and (c) increase in efficiency (10 times higher than conventional method) due to higher yield of positive hybridomas. In this method, biotinylated C-terminal peptide of PTMA was used as antigen and emulsified with complete Freund’s adjuvant. Rats were immunized once and 3 weeks later lymphocytes from the iliac lymph nodes of immunized rats were fused with SP2/0 myeloma cells. Antibody producing hybridomas were screened by ELISA and 16 positive clones were selected. On assaying reactivity of the purified antibody by western blotting and antibody testing for capacity to immunoprecipitate PTMA from tissue and the recombinant PTMA, it was evident that AntiCT antibody successfully immunoprecipitated PTMA from the cerebellum tissue lysate. When Anti N-terminal PTMA (2 F11) was applied alongside AntiCT for comparison and detection of PTMA immunoprecipitated by both antibodies carried out by 2 F11 using same amount of antibody for each antibody type; 2 F11 antibody showed greater capacity for immunoprecipitation than AntiCT. It is worth noting that the presence of variant proteins with the same N-terminal sequence generated from alternative splicing of the PTMA gene as recently reported [46] would possibly account for this difference. This observation needs to be verified in further prospective investigations.

The applied methodology revealed that the generated antibody was able to recognize full-length PTMA but could not detect the C-terminal truncated mutant as shown by the western blot results therefore, demonstrating the specificity of the antibodies to the C-terminal region of PTMA. Further characterization of the antibody by determining its cross reactivity with rat and human PTMA revealed clones with different species-specific affinities for PTMA while some clones lost the ability to secrete the antibodies possibly due to loss of chromosomes. Taken together, these results indicated successful generation of the C-terminal antibody. For subsequent experiments purified antibody from clone 7 (AntiCT) was used. A previous study by our laboratory showed that PTMA is a unique cell death regulatory molecule and therefore has neuroprotective roles in brain stroke [39, 45]. Additional studies demonstrated that PTMA exerts the neuroprotective function via its extracellular release upon interaction with S100A13 and that the C-terminal region of PTMA is required for this interaction [41]. However, PTMA cleavage by caspase in its C-terminus interferes with the interaction with S100A13 and disrupts its nuclear localization. Concurrently, PTMA undergoes asparaginyl endopeptidase-mediated cleavage in its N-terminus [42]. Such proteolytic modifications could present functional discontinuities of PTMA and aid to unearth other macromolecules that participate in PTMA functions. Therefore, we first investigated the presence of proteolytically modified variants of PTMA in mouse tissues by western blotting. When AntiCT was applied against 2 F11 in the immunoblot assay, a differential PTMA distribution pattern was observed in the brain regions and peripheral tissues. The tendency of PTMA expression pattern was similar by both antibodies, depicting a trend of high expression levels in brain regions associated with high neuron population (cerebellum) and regions in which neurogenesis is known to occur (olfactory bulb and hippocampus). Brain regions that showed the lowest levels include the thalamus and cortex. Only a single band signal was detectable, cleaved forms of PTMA were undetectable in brain region. In peripheral tissues PTMA expression was higher in lymphoid tissues such as thymus and spleen and lowest in the liver, only the thymus registered an additional lower band signal when detected by 2 F11 which was undetectable by AntiCT. It is plausible that this lower molecular weight band is indeed the C-terminal cleaved form of PTMA owing to the fact that the thymus is packed with apoptosing cells as a result of high cell turnover in this tissue. Further experiments will help to unequivocally determine the identity of the lower band. Overall, the expression level of cleaved forms of PTMA was undetectable in all tissues tested except the thymus under normal physiological context. It would be interesting to explore this phenomenon after induction of some stress. Following the revelation of the PTMA expression trend in the various tissues, we explored the subcellular localization by use of immunohistochemistry techniques.

Immunohistochemical analysis was performed by co-staining with both antibodies (AntiCT and 2 F11). Immunostaining with AntiCT revealed that in most of the tissues analyzed, the expression of PTMA was localized in both the nucleus and cytoplasm while PTMA immunoreactivity was mostly detected in cytoplasm or perinuclear space by 2 F11. Specific cells of the mucosal layers of duodenum, jejunum, ileum, colon and cecum corroborated this observation. Some cells in the submucosal layers of the duodenum and cecum showed PTMA immunoreactivity in the perinuclear space only by both antibodies. It is conceivable that this differential subcellular localization of PTMA is as a result of different PTMA interacting molecules in the different tissue and cell types or proteolytic modification of PTMA, however no PTMA cleaved forms were detected in these tissues. The brain hippocampus and stomach tissue sections revealed interesting staining patterns that differed from the aforementioned tissues. On one hand, exclusive nuclear staining of PTMA was observed in the hippocampus by both antibodies and on the other hand exclusive cytoplasmic staining was observed in the stomach when both antibodies were applied. As PTMA plays multiple cell robustness roles, this specified subcellular localization in the hippocampus and the stomach by both AntiCT and 2 F11 suggests cell type specific functioning of PTMA. The expression intensity in other tissues like the liver and the heart was relatively weak while the kidney showed comparatively higher signal intensity by immunohistochemistry where the signal was mainly localized in the nucleus. Interestingly the immunofluorescence detected in the lung tissues showed both nucleus and cytoplasmic staining by AntiCT but only cytoplasmic staining by 2 F11. This could be attributable to an interacting molecule involving the N-terminal of PTMA in the nucleus of lung cells. To determine the pattern of cellular distribution of PTMA in detail, immunocytochemistry analysis was carried out on primary neurons. As observed, AntiCT depicted a punctate distribution pattern in the nucleus of neurons and weakly stained the cytoplasm, features that differed from immunostaininng with 2 F11 which only stained the nucleus diffusely. We could consider the prospect that the punctate pattern detectable by AntiCT in primary neurons is a product of interacting molecules in the nucleus that mask C-terminal region and that the weak cytoplasmic signal detectable by AntiCT but not 2 F11, could indicate presence of interacting molecules in the cytoplasm that engage the N-terminal region of PTMA therefore masking it from detection by 2 F11. All together these expression patterns seem to suggest functional differences of PTMA in diverse tissues and cellular compartments likely associated with different interacting proteins. Under apoptotic conditions intact intracellular PTMA can be processed by proteases leading to different fragments that may differ in biological functions as showed by PTMA-S100A13 binding studies, it would therefore be significant to examine subcellular localization of PTMA under such varying conditions. Moreover, we cannot rule out possibility of the presence of PTMA variants with similar N-terminal regions but differing C-terminal compartments resulting from alternative splicing. Further prospective studies are required to confirm such possible effect.

## Conclusion

By analyzing how PTMA is distributed and localized in tissues and specific cell types under no pathological condition we have triggered prying into its precise role under such normal conditions given that in some tissues it was exclusively localized in the cytoplasm while in other tissues it was present predominantly in the nucleus. This will improve our understanding of mediators of PTMA biological function during disease. The next course of action would be to determine the particular cell types that express PTMA in the tissues that showed contrasting patterns of PTMA localization like the cells of the stomach, determine PTMA interacting partners and explore the biologic consequences of PTMA variants in same or different cells arising from alternative splicing.

## Additional files

Additional file 1:
**Densitometric quantification of the western blot bands of PTMA expression in the selected brain regions.** Each value is the Mean ± SD of at least three independent experiments. Arbitrary units were obtained by normalizing the signal to the internal control (values shown are PTMA signal/GAPDH signal). (DOCX 65 kb) Additional file 2:
**Densitometric quantification of the western blot bands of PTMA expression across peripheral tissues.** Each value is the Mean ± SD of at least three independent experiments. Arbitrary units were obtained by normalizing the signal to the internal control (values shown are PTMA signal/GAPDH signal). (DOCX 60 kb)

## Abbreviations

2 F11: antibody against the N-terminal region of prothymosin α
AntiCT: antibody against the C-terminal region of prothymosin α
CREB: cAMP response element binging protein
ECL: enhanced chemiluminescent
ELISA: Enzyme linked Immuno-sorbent Assay
HAT: Hypoxthanthine-Aminopterin-Thymidine
HRP: Horseradish Peroxidase
Keap1: Kelch-like ECH-associated protein 1
NLS: Nuclear Localization Signal
Nrf2: Nuclear Factor erythroid 2 related factor
OPD: O-phenylenediamine
PBS: Phosphate Buffered Saline
PEG: Polyethylene Glycol
PFA: Paraformaldehyde
PTMA: Prothymosin α
TA_1_: Thymosin Alpha_1_

## References

[1] Goldstein AL (2007). History of the discovery of the thymosins. Ann N Y Acad Sci.

[2] Haritos AA, Goodall GJ, Horecker BL (1984). Prothymosin alpha: isolation and properties of the major immunoreactive form of thymosin alpha 1 in rat thymus. Proc Natl Acad Sci U S A.

[3] Haritos AA, Tsolas O, Horecker BL (1984). Distribution of prothymosin alpha in rat tissues. Proc Natl Acad Sci U S A.

[4] Dosil M, Freire M, Gomez-Marquez J (1990). Tissue-specific and differential expression of prothymosin alpha gene during rat development. FEBS Lett.

[5] Enkemann SA, Ward RD, Berger SL (2000). Mobility within the nucleus and neighboring cytosol is a key feature of prothymosin-alpha. J Histochem Cytochem.

[6] Salvin SB, Horecker BL, Pan LX, Rabin BS (1987). The effect of dietary zinc and prothymosin alpha on cellular immune responses of RF/J mice. Clin Immunol Immunopathol.

[7] Barbini L, Gonzalez R, Dominguez F, Vega F (2006). Apoptotic and proliferating hepatocytes differ in prothymosin alpha expression and cell localization. Mol Cell Biochem.

[8] Mosoian A, Teixeira A, Burns CS, Khitrov G, Zhang W, Gusella L (2007). Influence of prothymosin-alpha on HIV-1 target cells. Ann N Y Acad Sci.

[9] Pineiro A, Cordero OJ, Nogueira M (2000). Fifteen years of prothymosin alpha: contradictory past and new horizons. Peptides.

[10] Gomez-Marquez J, Rodriguez P (1998). Prothymosin alpha is a chromatin-remodelling protein in mammalian cells. Biochem J.

[11] Hannappel E, Huff T (2003). The thymosins. Prothymosin alpha, parathymosin, and beta-thymosins: structure and function. Vitam Horm.

[12] Martini PG, Delage-Mourroux R, Kraichely DM, Katzenellenbogen BS (2000). Prothymosin alpha selectively enhances estrogen receptor transcriptional activity by interacting with a repressor of estrogen receptor activity. Mol Cell Biol.

[13] Tsai YS, Jou YC, Lee GF, Chen YC, Shiau AL, Tsai HT (2009). Aberrant prothymosin-alpha expression in human bladder cancer. Urology.

[14] Tzai TS, Tsai YS, Shiau AL, Wu CL, Shieh GS, Tsai HT (2006). Urine prothymosin-alpha as novel tumor marker for detection and follow-up of bladder cancer. Urology.

[15] Tsitsiloni OE, Stiakakis J, Koutselinis A, Gogas J, Markopoulos C, Yialouris P (1993). Expression of alpha-thymosins in human tissues in normal and abnormal growth. Proc Natl Acad Sci U S A.

[16] Dominguez F, Magdalena C, Cancio E, Roson E, Paredes J, Loidi L (1993). Tissue concentrations of prothymosin alpha: a novel proliferation index of primary breast cancer. Eur J Cancer.

[17] Magdalena C, Dominguez F, Loidi L, Puente JL (2000). Tumour prothymosin alpha content, a potential prognostic marker for primary breast cancer. Br J Cancer.

[18] Suzuki S, Takahashi S, Takahashi S, Takeshita K, Hikosaka A, Wakita T (2006). Expression of prothymosin alpha is correlated with development and progression in human prostate cancers. Prostate.

[19] Klimentzou P, Drougou A, Fehrenbacher B, Schaller M, Voelter W, Barbatis C (2008). Immunocytological and preliminary immunohistochemical studies of prothymosin alpha, a human cancer-associated polypeptide, with a well-characterized polyclonal antibody. J Histochem Cytochem.

[20] Wu CG, Habib NA, Mitry RR, Reitsma PH, van Deventer SJ, Chamuleau RA (1997). Overexpression of hepatic prothymosin alpha, a novel marker for human hepatocellular carcinoma. Br J Cancer.

[21] Tripathi SC, Matta A, Kaur J, Grigull J, Chauhan SS, Thakar A (2011). Overexpression of prothymosin alpha predicts poor disease outcome in head and neck cancer. PLoS One.

[22] Leys CM, Nomura S, LaFleur BJ, Ferrone S, Kaminishi M, Montgomery E (2007). Expression and prognostic significance of prothymosin-alpha and ERp57 in human gastric cancer. Surgery.

[23] Sasaki H, Fujii Y, Masaoka A, Yamakawa Y, Fukai I, Kiriyama M (1997). Elevated plasma thymosin-alpha1 levels in lung cancer patients. Eur J Cardiothorac Surg.

[24] Sasaki H, Nonaka M, Fujii Y, Yamakawa Y, Fukai I, Kiriyama M (2001). Expression of the prothymosin-a gene as a prognostic factor in lung cancer. Surg Today.

[25] Sasaki H, Sato Y, Kondo S, Fukai I, Kiriyama M, Yamakawa Y (2001). Expression of the prothymosin alpha mRNA correlated with that of N-myc in neuroblastoma. Cancer Lett.

[26] Ojima E, Inoue Y, Miki C, Mori M, Kusunoki M (2007). Effectiveness of gene expression profiling for response prediction of rectal cancer to preoperative radiotherapy. J Gastroenterol.

[27] Karetsou Z, Sandaltzopoulos R, Frangou-Lazaridis M, Lai CY, Tsolas O, Becker PB (1998). Prothymosin alpha modulates the interaction of histone H1 with chromatin. Nucleic Acids Res.

[28] Karetsou Z, Kretsovali A, Murphy C, Tsolas O, Papamarcaki T (2002). Prothymosin alpha interacts with the CREB-binding protein and potentiates transcription. EMBO Rep.

[29] Su BH, Tseng YL, Shieh GS, Chen YC, Shiang YC, Wu P (2013). Prothymosin alpha overexpression contributes to the development of pulmonary emphysema. Nat Commun.

[30] Karapetian RN, Evstafieva AG, Abaeva IS, Chichkova NV, Filonov GS, Rubtsov YP (2005). Nuclear oncoprotein prothymosin alpha is a partner of Keap1: implications for expression of oxidative stress-protecting genes. Mol Cell Biol.

[31] Niture SK, Jaiswal AK (2009). Prothymosin-alpha mediates nuclear import of the INrf2/Cul3 Rbx1 complex to degrade nuclear Nrf2. J Biol Chem.

[32] Jiang X, Kim HE, Shu H, Zhao Y, Zhang H, Kofron J (2003). Distinctive roles of PHAP proteins and prothymosin-alpha in a death regulatory pathway. Science.

[33] Markova OV, Evstafieva AG, Mansurova SE, Moussine SS, Palamarchuk LA, Pereverzev MO (2003). Cytochrome c is transformed from anti- to pro-oxidant when interacting with truncated oncoprotein prothymosin alpha. Biochim Biophys Acta.

[34] Malicet C, Giroux V, Vasseur S, Dagorn JC, Neira JL, Iovanna JL (2006). Regulation of apoptosis by the p8/prothymosin alpha complex. Proc Natl Acad Sci U S A.

[35] Evstafieva AG, Belov GA, Kalkum M, Chichkova NV, Bogdanov AA, Agol VI (2000). Prothymosin alpha fragmentation in apoptosis. FEBS Lett.

[36] Evstafieva AG, Belov GA, Rubtsov YP, Kalkum M, Joseph B, Chichkova NV (2003). Apoptosis-related fragmentation, translocation, and properties of human prothymosin alpha. Exp Cell Res.

[37] Enkemann SA, Wang RH, Trumbore MW, Berger SL (2000). Functional discontinuities in prothymosin alpha caused by caspase cleavage in apoptotic cells. J Cell Physiol.

[38] Skopeliti M, Iconomidou VA, Derhovanessian E, Pawelec G, Voelter W, Kalbacher H (2009). Prothymosin alpha immunoactive carboxyl-terminal peptide TKKQKTDEDD stimulates lymphocyte reactions, induces dendritic cell maturation and adopts a beta-sheet conformation in a sequence-specific manner. Mol Immunol.

[39] Ueda H, Fujita R, Yoshida A, Matsunaga H, Ueda M (2007). Identification of prothymosin-alpha1, the necrosis-apoptosis switch molecule in cortical neuronal cultures. J Cell Biol.

[40] Fujita R, Ueda H (2007). Prothymosin-alpha1 prevents necrosis and apoptosis following stroke. Cell Death Differ.

[41] Matsunaga H, Ueda H (2010). Stress-induced non-vesicular release of prothymosin-alpha initiated by an interaction with S100A13, and its blockade by caspase-3 cleavage. Cell Death Differ.

[42] Sarandeses CS, Covelo G, Diaz-Jullien C, Freire M (2003). Prothymosin alpha is processed to thymosin alpha 1 and thymosin alpha 11 by a lysosomal asparaginyl endopeptidase. J Biol Chem.

[43] Kishiro Y, Kagawa M, Naito I, Sado Y (1995). A novel method of preparing rat-monoclonal antibody-producing hybridomas by using rat medial iliac lymph node cells. Cell Struct Funct.

[44] Sado Y, Inoue S, Tomono Y, Omori H (2006). Lymphocytes from enlarged iliac lymph nodes as fusion partners for the production of monoclonal antibodies after a single tail base immunization attempt. Acta Histochemica Cytochemica.

[45] Ueda H, Matsunaga H, Halder SK (2012). Prothymosin alpha plays multifunctional cell robustness roles in genomic, epigenetic, and nongenomic mechanisms. Ann N Y Acad Sci.

[46] Teixeira A, Yen B, Gusella GL, Thomas AG, Mullen MP, Aberg J (2015). Prothymosin alpha variants isolated from CD8+ T cells and cervicovaginal fluid suppress HIV-1 replication through type I interferon induction. J Infect Dis.

[47] Aniello F, Branno M, De Rienzo G, Ferrara D, Palmiero C, Minucci S (2002). First evidence of prothymosin alpha in a non-mammalian vertebrate and its involvement in the spermatogenesis of the frog Rana esculenta. Mech Dev.

[48] Paolo Pariante, Raffaele Dotolo, Massimo Venditti, Diana Ferrara, Aldo Donizetti, Francesco Aniello and Sergio Minucci. Prothymosin alpha expression and localization during the spermatogenesis of Danio rerio. Zygote, available on CJO2015. doi:10.1017/S0967199415000568 10.1017/S0967199415000568 26450176

[49] Prisco M, Donizetti A, Aniello F, Locascio A, Del Giudice G, Agnese M (2009). Expression of Prothymosin alpha during the spermatogenesis of the spotted ray Torpedo marmorata. Gen Comp Endocrinol.

[50] Donizetti A, Liccardo D, Esposito D, Del Gaudio R, Locascio A, Ferrara D (2008). Differential expression of duplicated genes for prothymosin alpha during zebrafish development. Dev Dyn.

[51] Costopoulou D, Leondiadis L, Czarnecki J, Ferderigos N, Ithakissios DS, Livaniou E (1998). Direct ELISA method for the specific determination of prothymosin alpha in human specimens. J Immunoass.

