# Supplementary material for: Subcellular dissemination of prothymosin alpha at normal physiology: immunohistochemical vis-a-vis western blotting perspective
Source: BMC Physiol. 2016 Mar 1;16:2. doi: 10.1186/s12899-016-0021-4 (PMC4774093; doi:10.1186/s12899-016-0021-4)
Supplement: Additional file 2: — Densitometric quantification of the western blot bands of PTMA expression across peripheral tissues. Each value is the Mean ± SD of at least three independent experiments. Arbitrary units were obtained by normalizing the signal to the internal control (values shown are PTMA signal/GAPDH signal). (DOCX 60 kb) [file 12899_2016_21_MOESM2_ESM.docx]

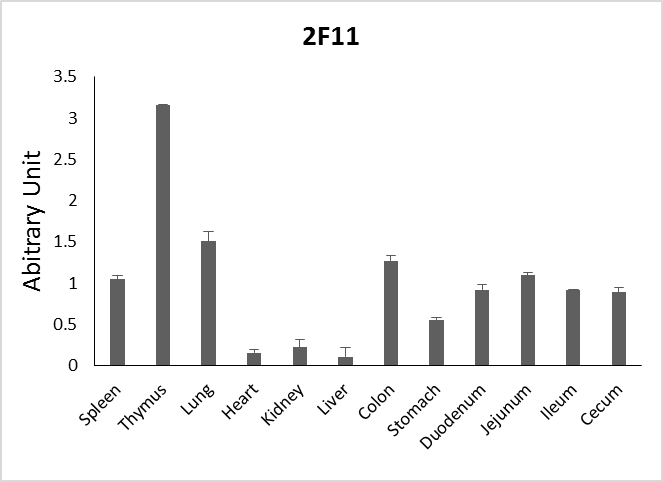

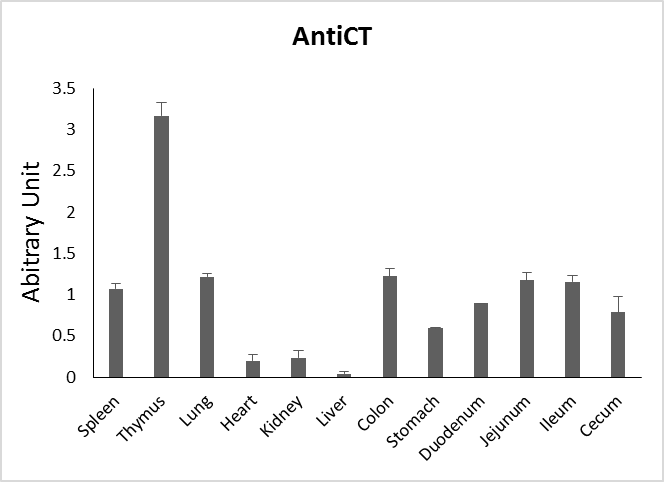


**Additional file 2. Densitometric quantification of the western blot bands of PTMA expression across peripheral tissues**.

Each value is the Mean ± SD of at least three independent experiments. Arbitrary units were obtained by normalizing the signal to the internal control (values shown are PTMA signal/GAPDH signal).
